# Supplementary material for: Mentha piperita L. Micropropagation and the Potential Influence of Plant Growth Regulators on Volatile Organic Compound Composition
Source: Molecules. 2020 Jun 7;25(11):2652. doi: 10.3390/molecules25112652 (PMC7321412; doi:10.3390/molecules25112652)
Supplement: Supplementary file 1 [file molecules-25-02652-s001.pdf]

1. Unknown compound - LRI 1021

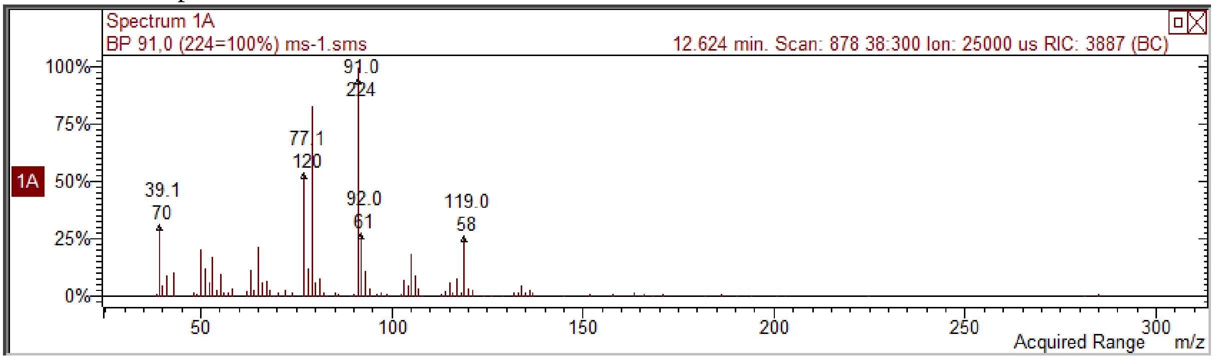

2. Unknown compound - LRI 1121

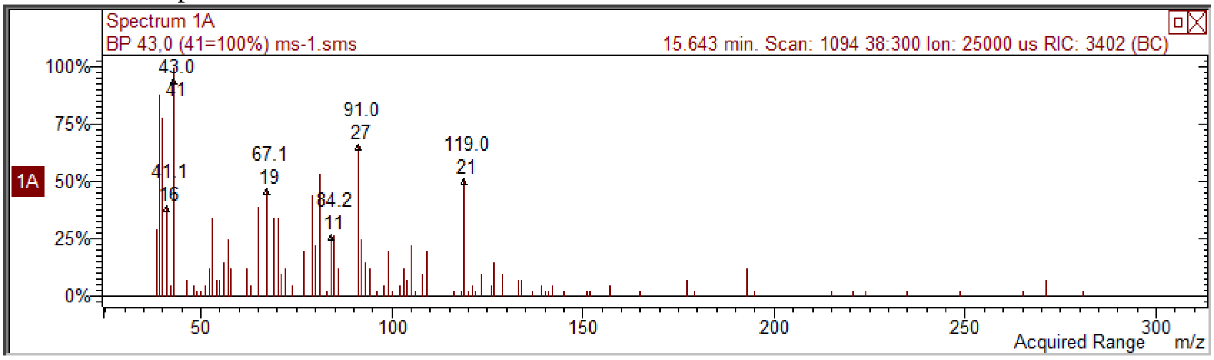

Figure 1 Mass spectra of unidentified VOCs in peppermint aroma profile. Both mass spectra was obtained by GC-MS analysis with Varian CP-3800/Saturn 2000 apparatus (Varian, Walnut Creek, CA, USA). MS operational conditions were as follows: ion source temperature: 250°C; electron impact (EI) ionization at 70 eV; scanning mode from 35 to 300 *m*/*z*.
